# Understanding the Biostimulant Action of Vegetal-Derived Protein Hydrolysates by High-Throughput Plant Phenotyping and Metabolomics: A Case Study on Tomato

**DOI:** 10.3389/fpls.2019.00047

**Published:** 2019-02-08

**Authors:** Kenny Paul, Mirella Sorrentino, Luigi Lucini, Youssef Rouphael, Mariateresa Cardarelli, Paolo Bonini, Hélène Reynaud, Renaud Canaguier, Martin Trtílek, Klára Panzarová, Giuseppe Colla

**Affiliations:** ^1^Photon Systems Instruments (PSI, spol.sr.o.), Drásov, Czechia; ^2^Department for Sustainable Food Process, Research Centre for Nutrigenomics and Proteomics, Università Cattolica del Sacro Cuore, Piacenza, Italy; ^3^Department of Agricultural Sciences, University of Naples Federico II, Naples, Italy; ^4^Centro di Ricerca Orticoltura e Florovivaismo, Consiglio per la Ricerca in Agricoltura e l’Analisi dell’Economia Agraria, Pontecagnano Faiano, Italy; ^5^NGAlab, Tarragona, Spain; ^6^Italpollina USA, Inc., Anderson, IN, United States; ^7^Nixe, Valbonne, France; ^8^Department of Agriculture and Forest Sciences, University of Tuscia, Viterbo, Italy; ^9^Arcadia Srl, Rivoli Veronese, Italy

**Keywords:** protein hydrolysates, integrative image-based high-throughput phenotyping, metabolomics, morpho-physiological traits, functional biostimulant characterization, ROS signaling

## Abstract

Designing and developing new biostimulants is a crucial process which requires an accurate testing of the product effects on the morpho-physiological traits of plants and a deep understanding of the mechanism of action of selected products. Product screening approaches using omics technologies have been found to be more efficient and cost effective in finding new biostimulant substances. A screening protocol based on the use of high-throughput phenotyping platform for screening new vegetal-derived protein hydrolysates (PHs) for biostimulant activity followed by a metabolomic analysis to elucidate the mechanism of the most active PHs has been applied on tomato crop. Eight PHs (A–G, I) derived from enzymatic hydrolysis of seed proteins of *Leguminosae* and *Brassicaceae* species were foliarly sprayed twice during the trial. A non-ionic surfactant Triton X-100 at 0.1% was also added to the solutions before spraying. A control treatment foliarly sprayed with distilled water containing 0.1% Triton X-100 was also included. Untreated and PH-treated tomato plants were monitored regularly using high-throughput non-invasive imaging technologies. The phenotyping approach we used is based on automated integrative analysis of photosynthetic performance, growth analysis, and color index analysis. The digital biomass of the plants sprayed with PH was generally increased. In particular, the relative growth rate and the growth performance were significantly improved by PHs A and I, respectively, compared to the untreated control plants. Kinetic chlorophyll fluorescence imaging did not allow to differentiate the photosynthetic performance of treated and untreated plants. Finally, MS-based untargeted metabolomics analysis was performed in order to characterize the functional mechanisms of selected PHs. The treatment modulated the multi-layer regulation process that involved the ethylene precursor and polyamines and affected the ROS-mediated signaling pathways. Although further investigation is needed to strengthen our findings, metabolomic data suggest that treated plants experienced a metabolic reprogramming following the application of the tested biostimulants. Nonetheless, our experimental data highlight the potential for combined use of high-throughput phenotyping and metabolomics to facilitate the screening of new substances with biostimulant properties and to provide a morpho-physiological and metabolomic gateway to the mechanisms underlying PHs action on plants.

## Introduction

Over the past decade, interest in plant biostimulants (PBs) has been on the rise, compelled by the growing interest of researchers, private industry and farmers in integrating these products in the array of environmentally friendly tools that secure improved crop productivity and yield stability under environmental stressors (Ertani et al., 2012, 2013; Haplern et al., 2015; Colla et al., 2017a; Yakhin et al., 2017; Rouphael et al., 2017a,c, 2018). Based on the new EU regulation, PBs are defined as ‘*CE marked products which stimulate plant physiological processes independently of the their nutrient content by improving one or more of the following characteristics of the plant rhizosphere or phyllosphere: nutrient use efficiency, tolerance to abiotic stress, crop quality, availability of confined nutrients in the soil and rhizosphere, humification and degradation of organic compounds in the soil*’ (European Commission, 2016). Protein hydrolysates (PHs) are an important category of PBs which are produced by chemical, enzymatic or by combining chemical and enzymatic hydrolysis of proteins from animal or plant source (Ertani et al., 2009, 2017; Niculescu et al., 2009; Calvo et al., 2014; Colla et al. 2015, 2016, 2017a,b). Over the past 10 years, plant-derived PHs produced through enzymatic hydrolysis have received huge interest from farmers due to their high agronomic value and the lack of limitation in their application on organically produced crops (Colla et al., 2014; Nardi et al., 2016). PH-based biostimulants can be applied to plants through foliar application or soil/substrate drenching. PHs sprayed in foliar way reach mesophyll cells by absorption through cuticle, epidermal cells and stomata (Fernández and Eichert, 2009) while in drench application, the absorption occurs through root epidermal cells and gets redistributed through xylem (Subbarao et al., 2015). PHs can also be applied as seed treatments especially for field crops such as wheat, corn, and soybean (Rouphael et al., 2018). PH application stimulates plant uptake of macro and micronutrients and helps in rapid plant growth and biomass accumulation, interfering with the carbon and nitrogen metabolic activities (Ertani et al., 2009, 2016; Colla et al., 2017a). PHs can also improve crop tolerance to abiotic stresses such as drought, salinity, and thermal stress (Ertani et al., 2013; Lucini et al., 2015; Colla et al., 2017a). Therefore, improving metabolic and physiological traits by PH-based biostimulant treatments provides novel strategies for maximizing biomass yield (Dudits et al., 2016). Development of highly effective PH-based biostimulants requires an accurate evaluation of the effects of candidate products on morpho-physiological traits of selected crops during different developmental stages and environmental conditions. As conventional screening methods are time consuming, destructive (e.g., fresh and dry weight estimation), labor intensive and expensive, high-throughput plant phenotyping procedures were recently proposed as effective and high-precision tools for product screening in order to increase the probability of finding new bioactive substances in a more cost- and time-effective manner (Povero et al., 2016; Rouphael et al., 2018; Ugena et al., 2018). ‘Phenomics’ as a technological tool considers systematic management of complex traits in genome (G) × environment (E) interactions (Houle et al., 2010). Plant phenotyping systems are fully automated robotic systems usually installed in a controlled environment or in semi-controlled greenhouse conditions. The phenotyping platforms are designed to ensure not only non-invasive monitoring of plants in throughput of few up to several hundreds of plants, but also provide means for automated cultivation and handling of the plants such as automated watering/weighing or nutrient delivery units (Fahlgren et al., 2015; Großkinsky et al., 2015). High-throughput phenotyping systems, which can capture plant growth, morphology, color and photosynthetic performance using RGB and chlorophyll fluorescence (ChlF) imaging tools, are highly promising and essential tools for dissecting physiological components in product screening and for dynamic quantitative analysis of plant growth and physiological performance (Rahaman et al., 2015; Awlia et al., 2016; Rouphael et al., 2018). RGB imaging is used to estimate the true color of each pixel and by using image processing algorithms for identification of plant-derived pixels. For identified plant objects, morphological and geometrical features are quantified including color properties (Rahaman et al., 2015). The pixel number-based assessment of plant volume or total leaf area correlates with fresh and dry weight of above ground plant biomass and can be thus used to evaluate green/fresh weight of the plants without cutting and measuring them (Fehér-Juhász et al., 2014; Fahlgren et al., 2015). Further image-based automated phenotyping permits time-series measurements that are necessary to follow the progression of growth performance and stress responses on individual plants.

Chlorophyll fluorescence is a popular technique in plant physiology used for rapid non-invasive measurement of photosystem II (PSII) activity. PSII activity is very sensitive to a range of biotic and abiotic factors and therefore the chlorophyll fluorescence technique is used as a rapid indicator of photosynthetic performance of plants in different developmental stages and/or in response to changing environment (Baker, 2008). The advantage of chlorophyll fluorescence measurements over other methods for monitoring stresses is that changes in chlorophyll fluorescence kinetic parameters often occur before other effects of stress are apparent (Murchie and Lawson, 2013). Chlorophyll fluorescence imagers integrated in high-throughput phenotyping platforms are becoming important tools for rapid screening for better photosynthetic performance and characterization of a plant’s ability to harvest light energy, which is directly related to plant biomass formation and plant architecture (Tschiersch et al., 2017).

Nonetheless, the comprehension of biochemical processes and physiological functions underlying the changes observed at phenotype level is of primary relevance to scientifically demonstrate and support the use of plant biostimulants, likely providing some clues on the best scenarios where these products can be used. It is expected that in the near future, provided that a regulatory framework will be implemented at least in the EU and United States, the information on mechanism/mode(s) of action will support biostimulants authorization and implementation. In this regard, metabolomics is being proposed as a close link between an organism’s genotype and phenotype (Lamichhane et al., 2018), including plant-environment interactions (Feussner and Polle, 2015). In fact, recent advances in metabolomics, data treatment and multi-variate statistics offer the possibility to achieve a rather inclusive phytochemical profile in biological systems, including plants, thus opening new opportunities (Meier et al., 2017; Tsugawa, 2018). This makes metabolomics a promising tool to elucidate, among others, the mode of action rather than the physiological processes involved in plant response to biostimulants.

Taking this background into consideration, the aim of this study was to unravel the morphological, physiological and biochemical mechanisms of action for protein hydrolysate-based biostimulants on tomato plants at early stage of growth (i.e., vegetative growth) by combining novel high-throughput plant phenotyping approach and metabolomics. Untreated and treated tomato plants were compared in terms of photosynthetic performance through kinetic chlorophyll fluorescence, and plant growth dynamics via RGB imaging by using high-throughput and non-invasive imaging technologies developed at Photon Systems Instruments (PSI, Czechia). Metabolomics analysis was performed to understand the mode of action of the best performing substances in improving plant growth. Evaluation of biostimulant activity at early growth stages of fruiting crops such as tomato can provide useful information for improving crop yield under field conditions. Crop traits like early vigor are associated with earliness of fruit maturity and high shoot biomass accumulation which have been often positively linked to increased yield of tomato crop (Kumar et al., 2015; Rouphael et al., 2017b). Finally, this study was also aimed to set up a methodology for screening plant biostimulants by combining an advanced phenotyping platform and metabolomic analysis.

## Materials and Methods

### Plant Material and Growing Conditions

**FIGURE 1:**
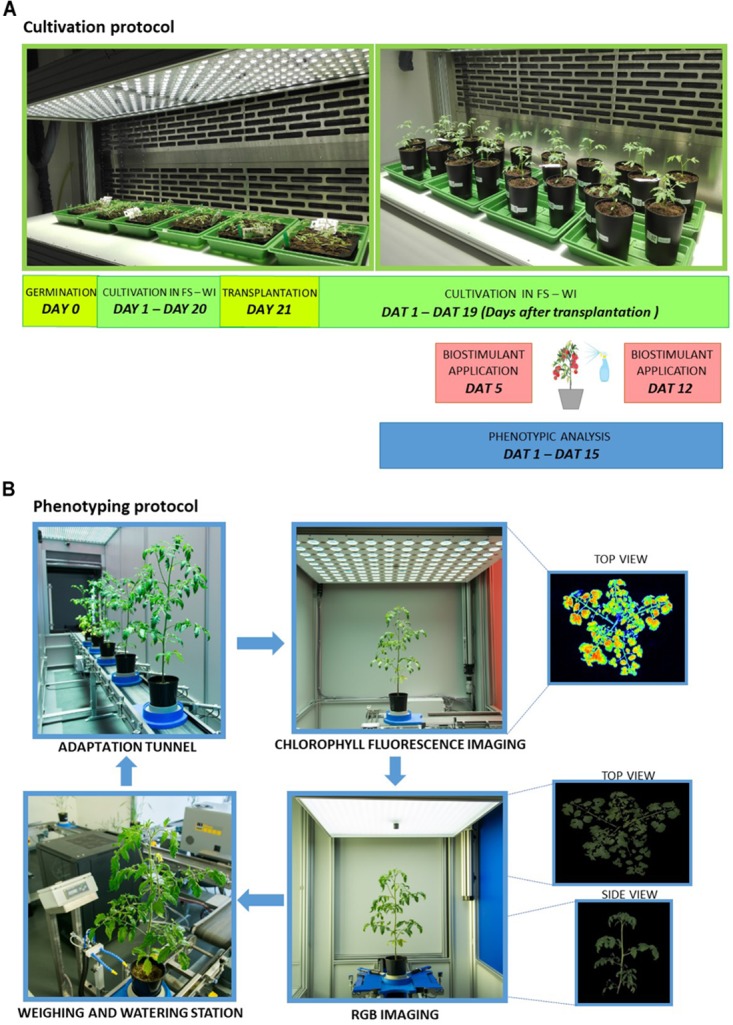
Schematic overview of cultivation protocol and automated phenotyping protocol. **(A)** Plants were cultivated for 20 days prior to transplantation in control conditions (FS-WI, PSI, Czechia) and were further kept in the same conditions for the next 19 days (DAT, days after transplantation). Eight types of protein hydrolysates (A–G, I) plus control treatment were applied twice to tomato plants by spraying 5 and 12 days after transplantation. Plant phenotypic measurements were performed during the experiment using PlantScreen^TM^ Modular System installed in semi-controlled greenhouse environment conditions in PSI Research Center (PSI, Drásov, Czechia). **(B)** Plant handling and automated phenotyping protocol. Tomato plants were transferred to PlantScreen^TM^ Modular System and automated phenotyping protocol was initiated. Prior to and following the protein hydrolysates application, plants were regularly screened using the calibrated top and side view RGB camera and kinetic chlorophyll fluorescence camera for photosynthetic performance quantification. Plants were regularly watered and weighed using the automated watering and weighing station.

Seeds of tomato (*Solanum lycopersicum* L. - Hybrid F1 Chicco Rosso) were sown in trays with 100 ml size of pots containing freshly sieved soil (Substrate 2, Klasmann-Deilmann GmbH, Germany) watered to full soil-water holding capacity. Trays with seeds were stratified for 2 days at 4°C in the dark. Trays were then transferred to a climate-controlled chamber (FytoScope FS_WI, PSI, Drásov, Czechia) with cultivation conditions set at 16 h day/8 h night regime, temperature set at 22°C day/20°C night, relative humidity set at 60% and light intensity set at 250 μmol photons m^-2^ s^-1^ for cool-white LED and 5.5 μmol photons m^-2^ s^-1^ for far-red LED lighting (Figure 1A).

### Plant Handling and Biostimulant Treatment

Prior initiation of automated phenotyping protocol, tomato plants were manually watered. Seven- and 14-day-old plants were watered to full saturation with fertilizers: 1.04 g L^-1^ calcium nitrate (15.5% N; 28% CaO), 0.04 g L^-1^ ammonium nitrate (34% N), 0.14 g L^-1^ monopotassium phosphate (52% P_2_O_5_, 34% K_2_O), 0.18 g L^-1^ potassium sulfate (50% K_2_O, 45%SO_3_), 0.5 g L^-1^ magnesium sulfate (10%N, 16% MgO), and 0.5 ml L^-1^ FloraMicro (5% N, 1% K_2_O, 5% Ca, 0.01% B, 0.001% Cu, 0.1% Fe, 0.05% Mn, 0.0008% Mo, 0.015% Zn).

Twenty-one-day-old plants reaching third true leaf stage were transplanted into 3 L pots filled with a mixture of Substrate 2 Klasmann soil and river sand in 3:1 ratio. Pots with soil mixture were prepared 1 day in advance of transplantation and were automatically watered in PlantScreen^TM^ Modular System to ensure soil moisture reaching 60% container capacity. For optimizing container capacity, one set of soil pots was dried for 3 days at 80°C and another set was saturated with water and left to drain for 1 day before weighing 100% water holding capacity (Awlia et al., 2016). Following transplantation, plants were regularly watered to defined reference weight (60% container capacity) automatically in PlantScreen^TM^ Modular System.

Plants were randomly distributed into nine groups with six biological replicates per group. Eight types of plant-derived protein hydrolysates (A–G, I) were provided by Italpollina Company (Rivoli Veronese, Italy). PHs were obtained by the advanced technology LISIVEG which is based on enzymatic hydrolysis of vegetal-derived proteins from different plant sources belonging to families of *Leguminosae* and *Brassicaceae*. Total nitrogen of each PH was as follow: 5.2% (A), 4.6% (B), 3.7% (C), 4.2% (D), 4.3% (E), 4.2% (F), 4.0% (G), 5% (I). PHs (A–G) were non-commercial products whereas I was a commercial biostimulant named ‘Trainer^®^’ derived from legume seeds. All PHs were foliarly sprayed in a water solution containing a non-ionic surfactant Triton X-100 at 0.1%. A control group sprayed with distilled water containing 0.1% Triton X-100 was also included. Foliage sprays were performed twice: 5 DAT (days after transplantation) referred to as Treatment 1 (T1) and 12 DAT referred to as Treatment 2 (T2). For 24 h prior to and post spraying, humidity in the cultivation chamber was kept at 85% relative humidity. For foliar spray treatments, 2 ml of given PH was diluted in 500 ml distilled water with 0.1% Triton X-100 and 60 ml of solution was applied by homogenous foliar spray over the entire plant surface per plant replica. Soil of each pot was covered with aluminum foil during and upon spraying and was removed prior to the next phenotypical analysis in PlantScreen^TM^ Modular System (Figure 1B). Right after foliar spray treatment, plants were taken back to fytoscope FS-WI.

### Phenotyping Protocol and Imaging Sensors

Plant phenotypic measurements were performed using PlantScreen^TM^ Modular System installed in semi-controlled greenhouse environment conditions in PSI Research Center (PSI, Drásov, Czechia). The platform was operated in a closed imaging loop that is within climatized environment with temperature ranging between 21 and 24°C. The platform is equipped with four robotic-assisted imaging units, automatic height measuring light curtain unit, an acclimation tunnel, and a weighing and watering unit. Plants set in individual transportation disks were transported to the individual units by a moving belt toward individual imaging and handling units. Twenty-two-day-old plants were randomly distributed into six batches, each batch containing 11 plants. Plant imaging started 1 DAT (day 1 of phenotyping) and continued until 15 DAT (day 15 of phenotyping). Plants were imaged using the following protocol. Briefly, plants were manually transferred from the climate-controlled growth chamber to the manual loading station of the PlantScreen^TM^ Modular System, transported to the acclimation tunnel through an automatic height measuring unit and dark adapted in an acclimation tunnel for 15 min prior to imaging. Successively, plants were automatically phenotyped for around 30 min per batch using kinetic chlorophyll fluorescence imaging measurement for photosynthetic performance analysis and top view and multiple angle side view Red Green Blue (RGB) imaging for morphological and growth analysis. Finally, plants were automatically transported to the watering and weighing unit for maintaining precise soil water holding capacity at 60%. After the end of the phenotyping protocol, plants were manually moved back to the climate-controlled growth chamber until the subsequent phenotyping day. Using the automatic timing function of PlantScreen^TM^ Scheduler (PSI, Drásov, Czechia), the phenotyping protocol was programmed to always start at the same time of the diurnal cycle (after 3 h of illumination in the climate-controlled growth chamber). Phenotyping protocol was recorded twice prior to biostimulant application in days 1 and 3 (pre-T measurements); three times post first biostimulant application in days 6, 8, and 10 (post T1 application) and twice post second biostimulant application in days 13 and 15 (post T2 application). The acquired images were automatically processed using Plant Data Analyzer (PSI, Drásov, Czechia) and the raw data exported into CSV files were provided as input for further analysis.

### Kinetic Chlorophyll Fluorescence Imaging

Kinetic chlorophyll fluorescence (ChlF) measurements were acquired using an enhanced version of the FluorCam FC-800MF pulse amplitude modulated (PAM) chlorophyll fluorometer (PSI, Czechia) (Awlia et al., 2016) with an imaging area in top view position of 800 mm × 800 mm as described in Tschiersch et al. (2017). Photosynthetic performance in the plants was assessed by quantifying the rate of photosynthesis at different photon irradiances using the light curve protocol (Henley, 1993; Rascher et al., 2000) which was proven to provide detailed information on ChlF under stress, information on photosynthetic performance in many studies dealing with plants’ stress and to quantify the rate of photosynthesis at different light irradiances (Digruber et al., 2018) (Supplementary Figure S1). Protocol described previously (Awlia et al., 2016) was optimized for the tomato plants from early to later developmental stage. Finally, three actinic light irradiances (Lss1- 170 μmol photons m^-2^ s^-1^, Lss2 – 620 μmol photons m^-2^ s^-1^, Lss3 - 1070 μmol photons m^-2^ s^-1^) with a duration of 30 s in the light curve protocol were used to quantify the rate of photosynthesis.

### Visible RGB Imaging

To assess digital biomass of the plants, RGB imaging was done from top view (RGB2) and side view from multiple angles (RGB1) (Supplementary Figure S2). The RGB imaging unit implemented in PlantScreen^TM^ Modular System is a light isolated box equipped with a turning table with precise angle positioning, two RGB cameras (top and side) mounted on robotic arms and each supplemented with LED-based lighting source to ensure homogenous illumination of the imaged object. Imaged area in top view position is 800 mm × 800 mm, imaged area from side view is 1205 mm × 1005 mm (height × width). Here we acquired side view images from three different angles (0, 120, and 240°) for side view RGB analysis. RGB images (resolution 2560 pixels × 1920 pixels) of the plants were captured using the GigE uEye UI-5580SE-C - 5 Megapixels QSXGA Camera with 1/2^′′^ CMOS Sensor (IDS, Germany) from top and side view. For side view projections, line scan mode was used with a resolution -2560 × 2956 px/scan, 200 lines per second. Lighting conditions, plant positioning and camera settings were fixed throughout the experiment. Raw RGB images were processed as described previously (Awlia et al., 2016) with some modifications for side view RGB image processing algorithms. Projected shoot area (PSA) for side view was calculated as average of plant specific pixels extracted from three side view images acquired from 0, 120, and 240° angles. PSA extracted from top and side view projections was used to estimate shoot biomass. Briefly side view and top view RGB images of the plants were used for calculation of plant volume, using the formula from Klukas et al. (2014):

$$V=\sqrt{A_{S(average)}^{2}\times A_{t}^{2}}$$

where *A*_s_ and *A*_t_ are the projected areas from side-view (at different angles) and top-view images, respectively. Volume was termed as “digital biomass,” as reported in a work from Rahaman et al. (2017). Digital biomass was used to calculate relative growth rate (RGR) between two timepoints T_1_ and T_2_ as follows:

$$RGR=(\ln\;W_{2}-\ln\;W_{1})/(T_{2}-T_{1})$$

In addition, height and width of the plants were calculated from the binary side view images. For shoot greenness evaluation, six hues of green were automatically generated using as input images all the original RGB images captured during the phenotyping period (DAT 1–DAT 15). These six most representative hues were selected and used to estimate the variations in shoot colors and are shown in RGB color scale as a percentage of the shoot area (pixel counts).

### Sample Harvest

Ninteen DAT (19th day of phenotyping) plant material was harvested. For metabolomic analysis of tomato plants treated with biostimulants A, B, I, and control plants third and fourth fully expanded leaves from the top of each plant were harvested. The non-commercial biostimulants A and B were selected for the metabolomic analysis based on the higher morpho-physiological traits and were compared to the commercial biostimulant (I) as well as to the untreated control treatment. Final biomass of each plant was determined by measuring fresh weight and dry weight of remaining shoot in a ventilated oven at 65°C until constant weight.

### Untargeted Metabolomics

Leaf samples (1.0 g) were extracted using an Ultra-Turrax (Ika T-25, Staufen, Germany), in 10 mL of 0.1% HCOOH in 80% aqueous methanol. The extracts were centrifuged (12,000 × *g*), then filtered through a 0.22 μm cellulose membrane directly into amber vials for analysis. Thereafter, untargeted metabolomics were carried out through an UHPLC chromatographic system coupled to a hybrid quadrupole-time-of-flight mass spectrometer (UHPLC/QTOF-MS). The metabolomic platform included a 1290 ultra-high-performance liquid chromatograph, a G6550 iFunnel Q-TOF mass spectrometer and a JetStream Dual Electrospray ionization source (all from Agilent technologies, Santa Clara, CA, United States). The analysis was carried out as previously described (Rouphael et al., 2016). Briefly, chromatographic separation was achieved in reverse phase mode, using an Agilent Zorbax Eclipse-plus C18 column (100 mm × 2.1 mm, 1.8 μm) and a linear gradient (5–95% methanol in water, 34 min run time) foe elution, with a flow of 220 μL min^-1^ at 35°C. The mass spectrometric acquisition was done in positive polarity and extended linear dynamic range SCAN (100–1000 *m/z*).

Features deconvolution and post-acquisition processing were done in Agilent Profinder B.06. After mass and retention time alignment, compounds annotation was achieved using the ‘find-by-formula’ algorithm based on monoisotopic accurate mass, isotopes spacing and isotopes ratio, with a mass accuracy tolerance of <5 ppm. The database PlantCyc 12.5 (Plant Metabolic Network^1^) was used for annotation purposes. Based on the strategy adopted, identification was carried out according to Level 2 (putatively annotated compounds) of COSMOS Metabolomics Standards Initiative^2^.

A filter-by-frequency post-processing filter was applied to retain only those compounds that were present in 75% of replications within at least one treatment. The classification of differential compounds into biochemical classes was carried following PubChem (NCBI^3^) and PlantCyc information.

### Data Management and Statistical Analysis

The data processing pipelines Plant Data Analyser (PSI, Drásov, Czechia) includes pre-processing, segmentation, feature extraction and post-processing. Values for projected shoot area were calculated from images taken in the visible light spectrum and correspond to volume estimation which were used as a proxy for the estimated biomass of the plants. Data were processed using MVApp application (mmjulkowska/MVApp: MVApp.pre-release_v2.0; Julkowska et al., unpublished). Using the MVApp, outliers were identified with the interquartile range rule as plants whose volume had a value 1.5 times away from the mean. Those plants were removed from the data set. Statistical differences between treatments and time points were determined by one-way analysis of variance (ANOVA) with *post hoc* Tukey’s Honest Significant Difference (HSD) test (*P*-value < 0.05) performed using appropriate scripts in MVApp tool. Data are displayed as mean ± standard deviation of the six independent plants per treatment.

Interpretation of metabolomic data was formerly carried out using Mass Profiler Professional B.12.06 as previously described (Salehi et al., 2018). Briefly, compound abundance was Log2 transformed and normalized at the 75th percentile and baselined against the median. Unsupervised hierarchical cluster analysis was formerly carried out using the fold-change based heatmap, setting similarity measure as ‘Euclidean’ and ‘Wards’ linkage rule. Thereafter, the dataset was exported in SIMCA 13 (Umetrics, Malmo, Sweden), UV-scaled and elaborated for Orthogonal Projections to Latent Structures Discriminant Analysis (OPLS-DA) modeling. This latter multivariate supervised statistic allowed separating variance into predictive and orthogonal (i.e., ascribable to technical and biological variation) components. Outliers were excluded using Hotelling’s T2 and adopting 95% and 99% confidence limits, for suspect and strong outliers, respectively. Model cross validation was done through CV-ANOVA (*p* < 0.01) and permutation testing (*N* = 300) was used to exclude overfitting. Model parameters (goodness-of-fit R^2^Y and goodness-of-prediction Q^2^Y) were also produced. Finally, Variable Importance in Projection (VIP) analysis was used to select the metabolites having the highest discrimination potential. A subsequent fold-change analysis was performed from VIPs to identify extent and direction of the changes in accumulation related to the biostimulants.

## Results and Discussion

### High-Throughput Phenotyping of Tomato Plant Growth

**FIGURE 2:**
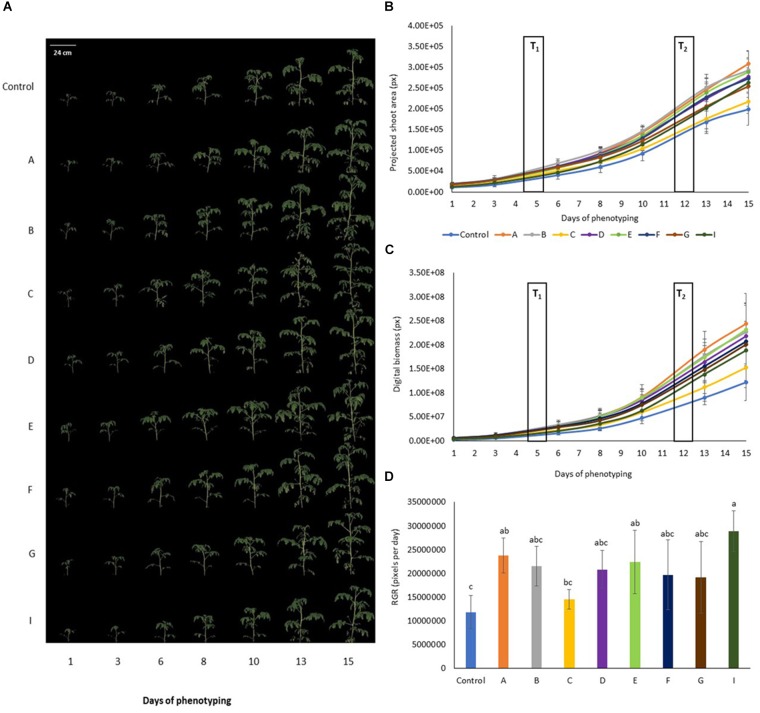
Color segmented side view Red Green Blue (RGB) images of tomato plants prior to and upon PHs application. **(A)** Side view (120°) RGB image of the tomato plants over the time of phenotyping period (D1–D15). **(B)** Projected shoot area over time of phenotyping period. Values represent the average of six biological replicates per treatment. Error bars represent standard deviation. T1 and T2 correspond to days of protein hydrolysates application by foliar spraying. **(C)** Digital biomass quantified over time of phenotyping period. Values represent the average of six biological replicates per treatment. Error bars represent standard deviation. T1 and T2 correspond to days of protein hydrolysate application by foliar spraying. **(D)** Comparison of relative growth rate for the different treatments quantified over phenotyping period following the protein hydrolysate treatments (DAT 6–DAT 15). Values represent the average of six biological replicates per treatment. Error bars represent standard deviation.

Visible light Red Green Blue (RGB) digital imaging based on using cameras sensitive in visible spectral range (400–750 nm) allows non-invasive dynamic quantification of shoot biomass, measurement of a wide range of plant morphological parameters and analysis of shoot color. Multiple angle side view images (Figure 2 and Supplementary Figure S2) and simple image stacks acquired from top view were used to calculate plant volume that is an approximate of digital biomass of the plants throughout the cultivation period. Regularly acquired multiple time points measurements were used to asses dynamic changes in plant morphology, color and calculate growth rates.

In general, tomato plants treated with PHs showed better shoot biomass production in comparison with the untreated control plants (Figure 2). Top view projected shoot area was increased in tomato plants treated with PHs A and E post first foliar treatment (Supplementary Table S1). For A treatment this correlated with PSA extracted from multiple angle side view RGB images (Supplementary Table S2) with B treatment improving the PSA in period between the two foliar treatments. In terms of morphological features extracted from both top and side view images such as compactness, height and width of the plants, treatments A, B, D, E and F gave an increase of height and width of plants (Supplementary Tables S3, S4). The digital biomass of the plants sprayed with PHs increased (Figure 2C), especially in the case of A treatment where the improved growth performance was significantly compared to untreated control plants from the 8th day of phenotyping, 3 days post first foliar spraying, respectively (Supplementary Table S5). The same trend was recorded when the growth dynamics was considered by evaluating plant growth rates. We quantified relative growth rates from DAT 6–DAT 15 representing growth performance of the plants following the two PH treatments that were applied on DAT 5 and DAT 12 (Figure 2D). For A, E and I treatment, growth rates were improved when compared to control plants, however, the effect of A and E treatment could not be discriminated from the effects of the other PHs. Interestingly, the treatment I was identified as the one with highest growth rate among all PHs. Overall among all treatments, the best growth performance trend in terms of biomass and growth rate was observed for tomato plants treated with treatment A, whereas tomato plants treated with PH named C were smaller with slower growth dynamics. This further correlated with analysis of dry and fresh weights of tomato shoots that were harvested following the end of the phenotyping period (*r* = 0.87^∗^ and 0.85^∗^ for fresh and dry weight, respectively).

We further evaluated the variation in shoot green colors over the phenotyping period by using greenness hue abundance automatically computed from color-segmented RGB images (Supplementary Figure S3). We calibrated the analysis algorithms by using RGB images from all treatments and all days of phenotyping as described previously in Awlia et al. (2016). Dynamic changes in green hues during the plant growth were observed, however, no significant differences in the green hues were detected between the treatments (Supplementary Table S6).

### High-Throughput Phenotyping of Photosynthetic Performance in Tomato Plants

Chlorophyll fluorescence imaging has become one of the most powerful and popular tools in plant biology for rapid non-invasive measurement of Photosystem II (PSII) activity. Because PSII activity is very sensitive to a wide range of stimuli, chlorophyll fluorescence imaging can be used as rapid indicator of plant photosynthetic performance in different developmental stages, and in response to environmental changes (Murchie and Lawson, 2013).

**FIGURE 3:**
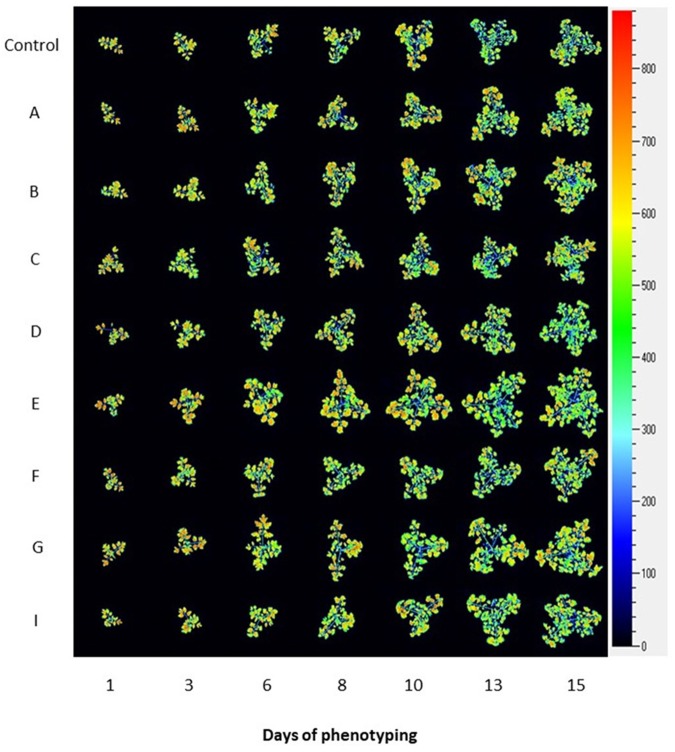
Photosynthetic performance of tomato plants visualized by kinetic chlorophyll fluorescence imaging in all protein hydrolysate treatments. Representative images of chlorophyll fluorescence for tomato plants prior to and upon PHs treatment. False-color images of maximum fluorescence value (FM) for tomato plants over phenotyping period (days 1–15) are shown.

To assess the physiological status of tomato plants treated with the biostimulants, we used the automated chlorophyll fluorescence imaging setup (Figure 3 and Supplementary Figure S1) and quantified the rate of photosynthesis at different photon irradiances using the light curve protocol (Henley, 1993; Rascher et al., 2000). From the measured fluorescence transient states, the basic ChlF parameters were derived (i.e., *F*_o_, *F*_m_, *F*_t_, and *F*_v_), which were used to calculate range of parameters characterizing plant photosynthetic performance (i.e., *F*_v_′/*F*_m_′, NPQ, qP, and ΦPSII) (for overview refer to Paul et al., 2011; Awlia et al., 2016; Tschiersch et al., 2017). In addition, ETR parameter was calculated which refers to photosynthetic electron transport rate of photosystem II and indicates the efficiency of linear electron flow route in the photosynthetic machinery for producing energy rich molecules ATP and NADPH.

**FIGURE 4:**
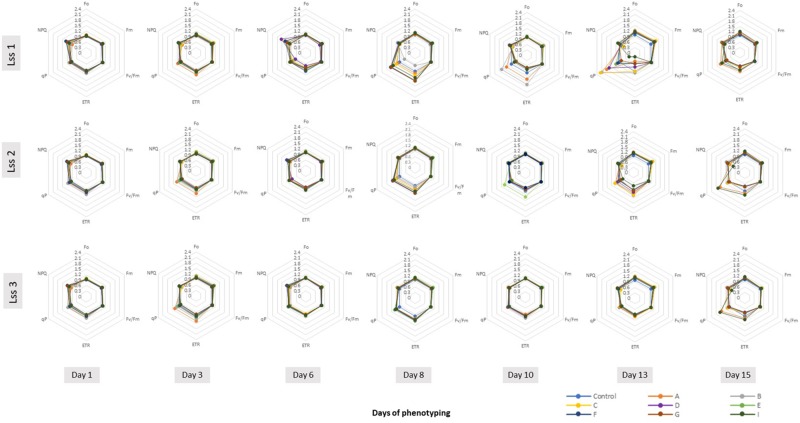
Spider plots of photosynthetic parameters deduced from kinetic chlorophyll fluorescence imaging on whole plant level in all treatments. Minimal fluorescence in dark-adapted state (*F*_o_), maximum fluorescence in dark-adapted state (*F*_m_), maximum quantum yield of PSII photochemistry for the light-adapted state (*F*_v_′/*F*_m_′), the photochemical quenching coefficient that estimates the fraction of open PSII reaction centers (qP), steady-state non-photochemical quenching (NPQ) and electron transport rate (ETR) were measured using the light curve protocol for tomato plants prior to and upon PHs treatments. The data are shown for the protein hydrolysate treated plants after normalization to respective values obtained in the control treatment at various time points of phenotyping period. Data are mean of six independent plants per treatment. Lss1, Lss2 and Lss3 represent actinic photon irradiance measurements taken at 170, 620, and 1070 μmol photons m^-2^ s^-1^, respectively.

We selected six of the parameters to characterize dynamically photosynthetic function of PSII in the tomato plants prior to and post biostimulant treatment: the minimal level of fluorescence measured in dark-adapted state (*F*_o_), the maximum level of fluorescence measured in dark-adapted state (*F*_m_), the maximum quantum yield of PSII photochemistry in the light-adapted state (*F*_v_′/*F*_m_′), the photochemical quenching coefficient that estimates the fraction of open PSII reaction centers (qP), steady-state non-photochemical quenching (NPQ) and PS II operating efficiency (ΦPSII) used for calculation of electron transport rate (ETR). ETR is a process correlated to the quantum yield of the CO_2_ assimilation mechanisms and to the overall photosynthetic capacity of the plants (Genty et al., 1989). As shown in Figure 4, the selected fluorescence parameters varied partially between the individual days following the PH treatment, however, we could not observe any trend among the treatments. In addition, we were not able to detect any significant changes in the ChlF parameters assessed (Supplementary Table S7). This was the case for all treatments at any photon irradiances used.

Kinetic chlorophyll fluorescence imaging used for non-invasive quantitative analysis of PSII fluorescence emission is especially suited to monitor physiological traits via changes in photochemistry. In the field of automated high-throughput phenotyping, PAM Chl fluorescence imaging is still not widely used in the imaging sensor platforms, however, a range of studies already demonstrated the broad potential of the technique to measure quantitatively physiological state of the plants and to diagnose the reactions of the plants to stress even before visible symptoms become apparent (Paul et al., 2011; Awlia et al., 2016; Tschiersch et al., 2017). Biostimulants have shown to increase photosynthetic efficiency, improve the efficiency of light utilization and dissipation of excitation energy in PSII antennae as well as an increase in photosynthetic pigments (Yakhin et al., 2017). The fact that in our case the application of the PHs did not result in improved photochemistry parameters, although the biomass of the biostimulant treated plants increased, might be associated with the beneficial action of PHs on stomatal conductance rather than on the PSII directly. This might improve net CO_2_ assimilation rate and consequently biomass production. Another putative mechanism involved in the stimulation of plant growth and productivity of PH-treated tomato plants could be the occurrence of smaller and more responsive stomata that are proposed to be able to sustain higher photosynthetic activities (Rouphael et al., 2017d).

### Metabolomics Analysis of Tomato Leaves for Understanding the Mode of Action of Selected PHs

A metabolomic approach was used, following phenotyping analysis, aimed to strengthen at the molecular level the effects of the PHs on morpho-physiological traits and plant growth. Indeed, the understanding of the mechanisms through which PHs act on a plant can effectively support their actual implementation into agricultural practices and possibly suggest specific contexts for their optimal and profitable use. With this aim, an untargeted UHPLC/QTOF-MS analysis was performed and multi variate statistics used to point out similarities/dissimilarities among metabolomic profiles of the PH-treated plants. The combination of a high-performance untargeted profiling, together with a rather comprehensive database (PlantCyc), resulted in a large dataset (overall, almost 1600 compounds annotated). A large chemical diversity was represented within the dataset, including compounds from a wide variety of biochemical classes and metabolic processes. The whole dataset, together with individual compounds’ abundance and composite mass spectra, is provided in supplementary material (Supplementary Table S8).

**FIGURE 5:**
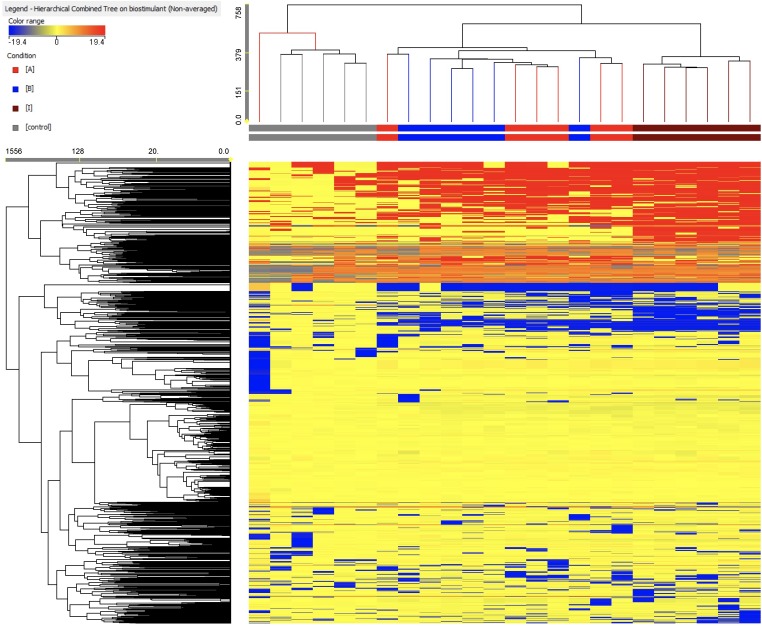
Unsupervised hierarchical cluster analysis carried out from metabolomic profiles following application of selected protein hydrolysates. Dendrograms were produced on the basis of fold-change heat-maps using metabolites profile gained from UHPLC-ESI/QTOF untargeted metabolomic profiling. The Wards’ linkage rule and Euclidean similarity were chosen to produce dendrograms.

As a first step of interpretation, a fold-change based hierarchical clustering was carried out (Figure 5). This unsupervised approach allowed producing two main clusters, one comprising all replications from the control and another including all PH-treated samples. In this latter, two further sub-clusters were evident, with products A and B being mixed together and with treatment I representing a separate sub-cluster. This unsupervised (i.e., naïve) classification of metabolomic profiles, based on individual fold-change values for each compound annotated, suggested that the PH treatments imposed a change in the plant metabolomic profile, and that treatments A and B induced a more comparable effect whereas treatment I had a more distinctive effect.

**FIGURE 6:**
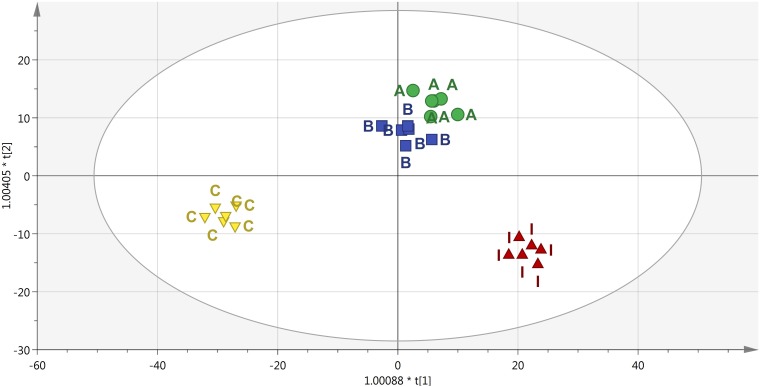
Score plot of Orthogonal Projection to Latent Structures Discriminant Analysis (OPLS-DA) supervised modeling carried out on metabolomic profiles following application of selected protein hydrolysates. The variation between groups was separated into predictive and orthogonal components (i.e., that ascribable to technical and biological variation). The OPLS model was cross-validated using CV-ANOVA (*p* < 0.01) and permutation tested to exclude over fitting. Furthermore, the presence of outliers was investigated according to Hotelling’s T2 method (i.e., the distance from the origin in the model) using 95 and 99% confidence limits for “suspect” and “strong” outliers, respectively. The pattern observed in the score plot was used to identify discriminant compounds based on Variable of Importance in projection (VIP) analysis.

To better identify the specific responses induced in plants following the PH treatments, a supervised OPLS-DA multivariate modeling was carried out. This discriminant analysis approach allows discriminating among groups into score plot hyperspace, by separating predictive and orthogonal components (i.e., those components ascribable to technical and biological variation) of variance. Looking at the OPLS-DA score plot (Figure 6), the outcome of this supervised approach was in agreement with hierarchical clusters. Indeed, the control clustered in a separate region of score plot hyperspace, treatment with products A and B were separated but still closer to each other, and treatment I was confirmed to have the most distinctive profile. The model parameters of the OPLS-DA regression were excellent, being R^2^Y and Q^2^Y 0.94 and 0.63, respectively. The model was validated (CV-ANOVA *P* = 0.009) and overfitting could be excluded through permutation testing (*N* = 100). Furthermore, the Hotelling’s T2 showed that suspect and strong outliers could be excluded. Given the more than acceptable model parameters, the variable selection method called VIP (Variable Importance in Projection) was used to explain the differences observed. The most discriminating compounds (i.e., the markers possessing a VIP score > 1.4) were exported and subjected to fold-change analysis against the control, to identify the trends of regulation altered by the treatments. The discriminant compounds, together with their VIP score and fold-change values, were grouped into chemical classes and are provided in Table 1. Interestingly, few biochemical classes included the most of discriminant metabolites. In more detail, low molecular weight phenolic compounds, poly-hydroxy fatty acids, membrane lipids (glyco- and phospholipids), hydroxy-carotenoids and phytohormones (polyamines) were the most represented.

**Table 1:** Discriminant metabolites as identified by variables of importance in projection (VIP) analysis following OPLS-DA modeling on metabolomic profile of treated plants.

Class	Metabolite	VIP score		[A] vs. [control]		[B] vs. [control]		[I] vs. [control]	
					
		Score	*SE*	Log FC	Regulation	Log FC	Regulation	Log FC	Regulation
Phenolics	3.5-dihydroxyanisole	1.409	0.769						
	1.3.5-trimethoxybenzene	1.405	0.286	2.8	Up	5.5	Up	1.7	Up
	4-hydroxybenzaldehyde	1.418	r0.820						
	3.6.7.4′-tetramethylquercetagetin 3′-*O*-beta-D-glucoside	1.540	r0.883						
	3-phenylpropanoate	1.457	0.308	0.3	Up	5.5	Up	1.7	Up
	3-hydroxybenzaldehyde	1.418	0.820						
	Gallocatechin	1.372	0.548	0.2	Up	3.6	Up	4.5	Up
	Leucocyanidin	1.372	0.548	0.2	Up	3.6	Up	4.5	Up
	Epigallocatechin	1.372	0.548	0.5	Up	3.6	Up	4.3	Up
Glucosinolates	3-(7′-methylthio) heptylmalate	1.308	0.304	3.1	Up	1.2	Up	1.8	Up
	2-(7’-methylthio) heptylmalate	1.308	0.304	3.7	Up	1.2	Up	1.8	Up
Lipids	Oleate	1.367	0.497	-29.4	Down	0.2	Up	3.9	Up
	Colneleate	1.515	0.219	-3.9	Down	-4.0	Down	2.0	Up
	4-coumaryl alcohol	1.456	0.313	0.3	Up	5.5	Up	1.7	Up
	Germacra-1(10).4.11(13)-trien-12-ol	1.315	0.777	-8.7	Down	-5.1	Down	0.4	Up
	Dammarenediol II	1.428	0.899	6.2	Up	6.2	Up	6.0	Up
	1-16:0-2-18:3-diacylglycerol-trimethylhomoserine	1.365	0.919	1.0	Up	1.1	Up	0.7	Up
	1-16:0-2-18:2-digalactosyldiacylglycerol	1.394	1.122						
	Sitosterol	1.317	1.095	-0.5	Down	-1.1	Down	-0.5	Down
	(12Z.15Z)-9.10-epoxyoctadeca-12.15-dienoate	1.515	0.219	-3.9	Down	-4.0	Down	2.0	Up
	An epoxy-octadeca-dienoate	1.515	0.219	-3.9	Down	-4.0	Down	2.0	Up
	A dihydroxyoctadeca-dienoate	1.371	0.724	0.6	Up	-0.4	Down	1.5	Up
	9.10-12.13-diepoxyoctadecanoate	1.316	0.617	11.6	Up	1.2	Up	6.9	Up
	16-alpha-hydroxygypsogenate-28-beta-D-glucoside	1.319	0.684	0.6	Up	8.8	Up	1.5	Up
	2-hydroxyhexadecanoate	1.413	0.883						
	2-*trans*-6-*trans*-farnesyl monophosphate	1.397	0.571	4.5	Up	4.6	Up	0.7	Up
	Geranyl monophosphate	1.376	0.378	3.2	Up	3.1	Up	1.6	Up
	(9S)-HPODE/(13S)-HPODE	1.371	0.724	0.6	Up	-0.4	Down	1.5	Up
	3–beta;-D-galactosyl-sn-glycerol	1.369	1.015						
	A 2-acyl-sn-glycero-3-phosphoethanolamine (n-C14:1)	1.357	0.447	3.1	Up	9.4	Up	1.9	Up
	A 1-acyl-sn-glycero-3-phosphoglycerol (n-C14:1)	1.346	0.288	-0.4	Down	-0.4	Down	-1.8	Down
	3.4-dihydroxy-5-iall-trans/i-hexaprenylbenzoate	1.323	0.679	-3.1	Down	9.3	Up	6.1	Up
	4.4-dimethyl-5-alpha-cholest-7-en-3-beta-ol/4.4-dimethyl-5-alpha-cholesta-8-en-3-beta-ol	1.317	1.095	-0.5	Down	-1.1	Down	-0.5	Down
	1.2-dipalmitoyl-phosphatidylglycerol-phosphate	1.317	0.506	-9.4	Down	-7.5	Down	1.4	Up
	(6E)-8-oxogeranial	1.315	0.666	-1.8	Down	-1.8	Down	-1.5	Down
	(2E.6E)-farnesal	1.315	0.777	-8.7	Down	-5.1	Down	0.4	Up
	4-alpha-carboxy-4-beta-methyl-5-alpha-cholesta-8-en-3-beta-ol	1.312	0.670						
Carotenoids	4-methylocta-2.4.6-trienedial	1.456	0.313	0.6	Up	5.5	Up	1.7	Up
	5.6-epoxy-3-hydroxy-5.6-dihydro-12′-apo-beta;-caroten-12′-al	1.500	0.534	-0.28646278	Down	-1.0	Down	-1.3	Down
	18′-hydroxy-chi; chi;-caroten-18-oate	1.304	0.646	-9.2	Down	-1.5	Down	-1.5	Down
Hormones	1-aminocyclopropane-1-carboxylate	1.419	0.241	2.9	Up	2.9	Up	1.8	Up
	Salicylaldehyde	1.418	0.820						
Others	Triferuloyl spermidine	1.503	0.350	0.6	Up	2.8	Up	1.7	Up
	Sinapoyltyramine	1.516	0.366	0.6	Up	-0.4	Down	1.8	Up
	Thiamin	1.450	0.539	4.6	Up	4.5	Up	0.4	Up
	*S*-adenosyl 3-(methylthio) propylamine	1.431	0.440	-6.1	Down	9.2	Up	6.1	Up
	Methyl-1.4-benzoquinone	1.418	0.820						
	*N*-acetylneuraminate	1.384	0.363	-1.4	Down	-1.5	Down	-1.5	Down
	Menaquinol-8	1.367	0.491						
	Pyropheophorbide *a*	1.361	0.302	-1.1	Down	-1.0	Down	-1.2	Down


*Discriminant metabolites (VIP > 1.4) are provided together with individual scores, their standard error (SE) and metabolite fold-change (FC) Log values, as compared to control; missing values denote fold-change values < 1.5.*

From an overall perspective, the metabolomic changes imposed by the PH treatment can be correlated to relatively few processes, all of them converging toward the ROS-related plant signaling network. Among plant growth regulators, 1-aminocyclopropane-1-carboxylate (ACC), i.e., the direct precursor of ethylene, was found up accumulated in treated plants. Considering that ethylene is not detectable by our metabolomic approach, the increase of ACC suggests and increase in ethylene itself. The effects of ethylene on growth and development have been found to vary, depending on other phytohormone profile, CO_2_ and light (Small and Degenhardt, 2018). Although usually related to senescence and fruit ripening, ethylene has been reported to play many other regulations in plants, including flowering and overall plant growth, cell division and root initiation, as well as modulation of secondary metabolites light (Schaller, 2012; Small and Degenhardt, 2018). In fact, at relatively low concentration, ethylene has been reported to stimulate leaf growth (Dubois et al., 2018) and to promote yields (Habben et al., 2014). Scientific evidence suggests that such ethylene-dependent regulation of plant growth is related redox signaling pathways (Caviglia et al., 2018).

Notably, polyamine conjugates (namely sinapoyltyramine and triferuloyl spermidine, both up accumulated in treated plants) were additional plant growth regulators being induced by the treatments. Polyamines are preferentially detected in actively growing tissues and have been implicated in the control of cell division, embryogenesis, root formation, fruit development and ripening, and responses to biotic and abiotic stresses (Kumar et al., 1997; Gill and Tuteja, 2010; Agudelo-Romero et al., 2013; Rouphael et al., 2016). However, these metabolites are also reported to affect H_2_O_2_ signature under salt stress (Gémes et al., 2017) in a coordinate manner with ethylene (Hou et al., 2013).

Even though a clear trend could not be observed, a wide alteration of the profile of membrane lipids was observed in our experiments. Such modulation might be the consequence of the altered signature in signaling compounds and antioxidants. Nevertheless, it is important to consider that membrane lipids play an important role in secondary signaling cascades which control plant adaptation processes (Hou et al., 2016). The concurrent changes in antioxidant compounds such as phenolics and carotenoids, suggests a fine tuning of the ROS-mediated signaling in tomato following application of the biostimulants. Indeed, such secondary metabolites are well known to play a pivotal role in plant defense against oxidative stress (Shalaby and Horwitz, 2015; Lucini et al., 2018; Rouphael et al., 2018). Such interplay between polyamines, ROS and ethylene was reported to alleviate the decrease of plant biomass under stress conditions (Gémes et al., 2017) and might have had a role also in our experiments. Consistently with our findings, it is interesting to note that such support to biomass accumulation was not related to photosynthetic efficiency (Gémes et al., 2017) and was linked to the accumulation of phenolic compounds (Gémes et al., 2016).

Unlike mammals, plants produce the most of ROS in chloroplast, under a controlled multi-level antioxidant-scavenging system that includes thiols, antioxidant enzymes and low molecular weight antioxidants to manage their accumulation and transmit oxidative signals. While the concept that deleterious and irreversible oxidation driven by ROS is embed in literature, the scientific consensus is now shifting toward the recognition of the positive roles of ROS as essential components of chloroplast-nucleus retrograde signaling pathways (Foyer et al., 2017; Foyer, 2018). Since H_2_O_2_ is relatively more stable than superoxide and singlet oxygen (both having short half-lives), this compound is believed the likely candidate to diffuse over any distances within the cell. Such redox signals interact with the phytohormone signaling network to regulate plant growth and defense processes (Foyer, 2018). This production of ROS is essential not only to convey communication regarding the redox pressure within the electron transport chain, but also to trigger short-term genetic responses (Foyer, 2018).

Within this redox-mediated multi-layer signaling process, carotenoids (together with glutathione and tocopherols) are among the most effective ^1^O_2_ scavengers; in fact, alteration in carotenoid oxidized forms has been recorded in our experiments. Coherently, the down accumulation of pheophorbide *a*, i.e., a precursor of chlorophylls, is a known process plant uses to control ROS production in the photosynthetic organs, given the fact that the photoreduction of oxygen to the superoxide radical is related to a reduced electron transport in PSI (Ghandchi et al., 2016) Although the link between the application of our PHs and biostimulants activity tomato could not be fully elucidated, a general consensus toward ROS-phytohormone interplay can be postulated, based on the differential metabolites identified by metabolomics. Such multi-level signaling might have played a role in determining the differences in growth observed through phenotyping.

## Conclusion

The use of PBs in particular vegetal-derived protein hydrolysates (PHs) in agriculture has greatly increased in the last decade mostly due to their *multifaceted properties*. Highly efficient and effective PH-based biostimulant products can be obtained using the ‘omics’ sciences. A novel approach based on the use of high-throughput phenotyping technologies and metabolomics was successfully tested on tomato crop for identifying new PHs with biostimulant activity and for studying the PH effects on plants at metabolic level. Dynamic monitoring of plant performance by high-throughput phenotyping system has proven to be a powerful tool for substance screening on the basis of morpho-physiological traits quantification. The effects of PHs on tomato phenotype were more evident on digital biomass. Metabolomics followed by multivariate analysis allowed elucidating the metabolic signatures imposed by the specific PH treatments. PH treatments affected the metabolic profile of tomato leaves via the modulation of a complex signaling process that involved the direct precursor of ethylene and polyamine conjugates. The coordinated action of plant growth regulators together with antioxidant compounds such as carotenoids and phenolics, might have affected the ROS-mediated signaling pathways. Although further assays under defined conditions would strengthen our findings, the discriminant compounds pointed out by this approach suggest that treated plants might experience a metabolic reprogramming following the application of the tested biostimulants.

## Author Contributions

KP wrote the first draft of the manuscript and followed the phenotyping measurements and data interpretation. MS performed the big data analysis. LL and PB performed the metabolomics analysis, data interpretation, and wrote the metabolomic part. YR, MC, HR, RC, and MT were involved in data analysis, data interpretation, and writing. GC and KLP coordinated the whole project, provided the intellectual input, set up the experiment, and corrected the manuscript.

## Conflict of Interest Statement

MT is the owner and CEO of PSI (Photon Systems Instruments), Drásov, Czechia, and KLP is an employee of his company. KP is an ex. employee of PSI and MS is a Ph.D. student, both conducted the experiments at PSI. RC is the Director of Nixe Company. HR is an employee of Italpollina Company (Anderson, United States) who provided the eight types of plant-derived protein hydrolysates (A–G, I). GC is a member of the spin-off Company Arcadia Srl approved by University of Tuscia, Italy. The remaining authors declare that the research was conducted in the absence of any commercial or financial relationships that could be construed as a potential conflict of interest.

## Footnotes

1 http://www.plantcyc.org

2 http://cosmos-fp7.eu/msi.html

3 https://pubchem.ncbi.nlm.nih.gov/

## Supplementary Material

FIGURE S1 Schematic of the kinetic ChlF protocol in the PlantScreen^TM^ Modular System. ChlF kinetics were captured with a PAM-based chlorophyll fluorometer. Images of the individual transient states were recorded. Corresponding frames were averaged for the measured parameters (*F*_o_, *F*_m_, *F*_m_′, *F*_t_, and *F*_p_) or calculated from the captured frames to compute the relative parameters such as *F*_v_/*F*_m_, ΦPSII, *F*_v_′/*F*_m_′, NPQ, and others. Automated ChlF image processing consisted of image segmentation by mask application, background subtraction and feature extraction. The signals from all pixels of each segment were averaged at each given time point. MF refers to the measuring flash, and yellow arrows indicate the saturation pulses that transiently saturated the electron transport chain. Lss1, Lss2, and Lss3 represent actinic photon irradiance measurements taken at 170, 620, and 1070 μmol photons m^-2^ s^-1^, respectively.

FIGURE S2 Schematic of top and side view RGB image processing. Original RGB images were automatically processed using the PlantScreen^TM^ Analyzer software to correct for barrel distortion caused by the fisheye lens, subtract the background and crop to isolate the plants within the imaged area, producing a binary (black and white) image. The binary images represent the plant surface (white) and background (black). Non-plant pixels, such as pots, were automatically removed to extract only plant pixels. Morphological analysis was conducted after separating the background from the plant shoot tissue. To evaluate color of plant shoot, RGB images were color-segmented to extract the green hues.

FIGURE S3 Variation in shoot colors of tomato plants. Dynamic relative changes in greenness hue abundance over the phenotyping period in control tomato plants and plants treated with protein hydrolysates (A–G, I). The six most representative color hues are shown in RGB color scale as percentage of the shoot area (pixel counts) of six biological replicates per treatment.

TABLE S1 Projected shoot area (PSA) of the tomato plants extracted from top view RGB images starting 3 days after the first PH application (day after transplanting, DAT = 8). Values are expressed as number of green pixels and represent the average of six biological replicates per treatment ± standard deviation. Within the same row and for the specified day different letters indicate significant difference according to one-way ANOVA *post hoc* Tukey’s test (*p* < 0.05).

TABLE S2 Projected shoot area (PSA) of the tomato plants extracted from multiple side view RGB images starting 3 days after the first PH application (day after transplanting, DAT = 8). Values are expressed as number of green pixels and represent the average of six biological replicates per treatment ± standard deviation. Within the same row and for the specified day different letters indicate significant difference according to one-way ANOVA *post hoc* Tukey’s test (*p* < 0.05).

TABLE S3 Width of the tomato plants extracted from multiple side view RGB images starting 3 days after the first PH application (day after transplanting, DAT = 8). Values are expressed as number of green pixels and represent the average of six biological replicates per treatment ± standard deviation. Within the same row and for the specified day different letters indicate significant difference according to one-way ANOVA *post hoc* Tukey’s test (*p* < 0.05).

TABLE S4 Height of the tomato plants extracted from multiple side view RGB images starting 3 days after the first PH application (day after transplanting, DAT = 8). Values are expressed as number of green pixels and represent the average of six biological replicates per treatment ± standard deviation. Within the same row and for the specified day different letters indicate significant difference according to one-way ANOVA *post hoc* Tukey’s test (*p* < 0.05).

TABLE S5 Digital biomass of tomato plants treated with different protein hydrolysates starting 3 days after the first PH application (day after transplanting, DAT = 8). Values are expressed as number of green pixels and represent the average of six biological replicates per treatment ± standard deviation. Within the same row and for the specified day different letters indicate significant difference in digital biomass, according to one-way ANOVA *post hoc* Tukey’s test (*p* < 0.05).

TABLE S6 Variation in shoot colours of tomato plants treated with different protein hydrolysates at 15 days after transplanting. The values for 6 most representative colour hues are shown as percentage of the shoot area (pixel counts). Values represent the average of six biological replicates per treatment ± standard deviation. Within the same row and for the specified day different letters indicate significant difference according to one-way ANOVA *post hoc* Tukey’s test (*p* < 0.05).

TABLE S7 Photosynthetic performance of tomato plants at 15 days after transplanting. Photosynthetic parameters deduced from kinetic chlorophyll fluorescence imaging on whole plant level in all protein hydrolysate treatments. Minimal fluorescence in dark-adapted state (*F*_o_), maximum fluorescence in dark-adapted state (*F*_m_), maximum quantum yield of PSII photochemistry for the dark-adapted (*F*_v_/*F*_m_), the photochemical quenching coefficient that estimates the fraction of open PSII reaction centers (qP), steady-state non-photochemical quenching (NPQ) and electron transport rate (ETR) were measured using the light curve protocol for tomato plants prior and upon PHs application. Values represent the average of six biological replicates per treatment ± standard deviation. Within the same row and for the specified day different letters indicate significant difference according to one-way ANOVA *post hoc* Tukey’s test (*p* < 0.05). Lss1, Lss2, and Lss3 represent actinic photon irradiance measurements taken at 170, 620, and 1070 μmol photons m^-2^s^-1^ PAR values, respectively.

TABLE S8 List of compounds identified by UHPLC-ESI QTOF-MS metabolomics in tomato plants (Level 2 of COSMOS standards - http://cosmos-fp7.eu/msi.html).
