# Supplementary figures and images for: Understanding the Biostimulant Action of Vegetal-Derived Protein Hydrolysates by High-Throughput Plant Phenotyping and Metabolomics: A Case Study on Tomato
Source: Front Plant Sci. 2019 Feb 8;10:47. doi: 10.3389/fpls.2019.00047 (PMC6376207; doi:10.3389/fpls.2019.00047)

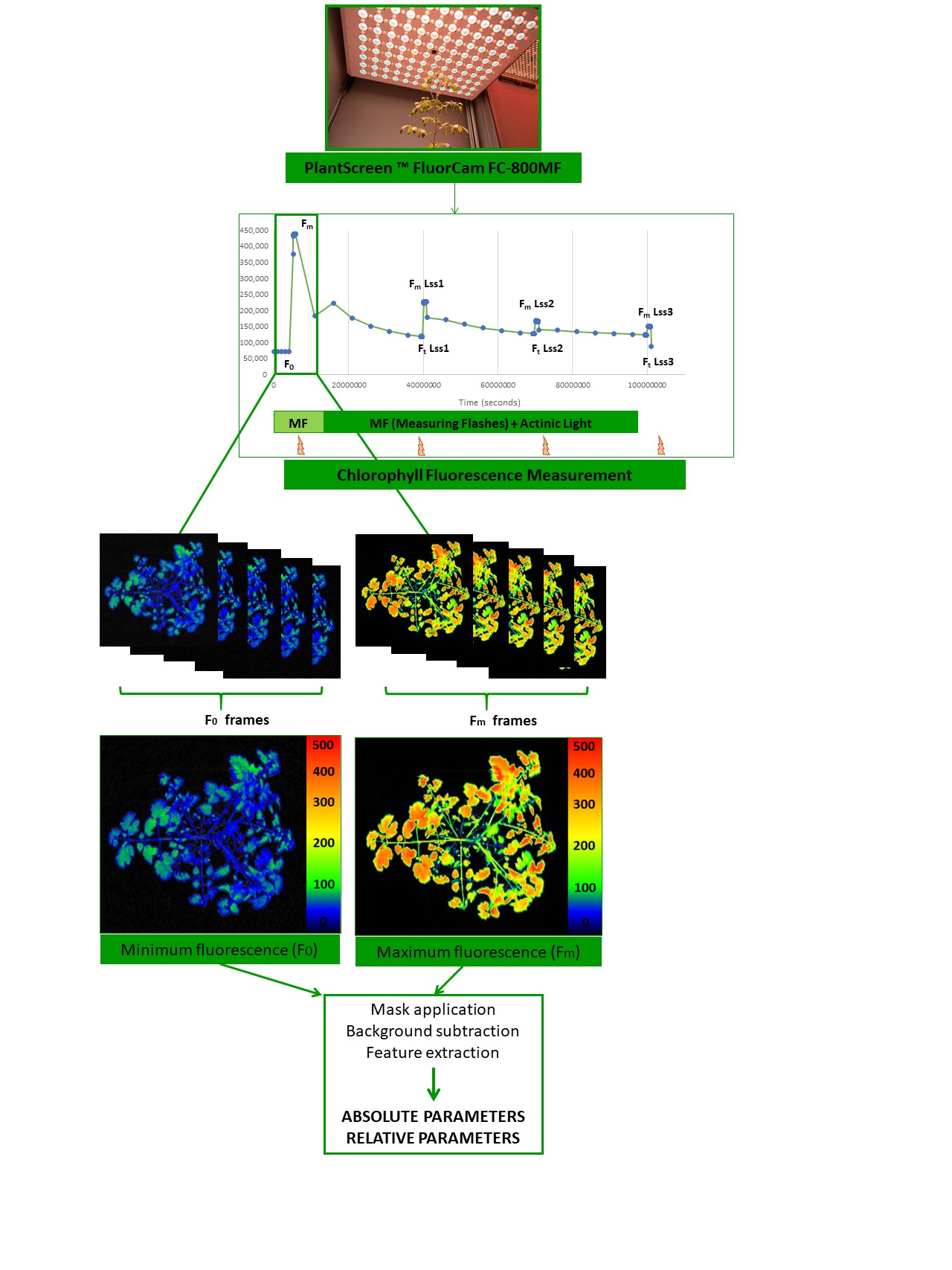

Supplement: FIGURE S1 — Schematic of the kinetic ChlF protocol in the PlantScreenTM Modular System. ChlF kinetics were captured with a PAM-based chlorophyll fluorometer. Images of the individual transient states were recorded. Corresponding frames were averaged for the measured parameters (Fo, Fm, Fm′, Ft, and Fp) or calculated from the captured frames to compute the relative parameters such as Fv/Fm, ΦPSII, Fv′/Fm′, NPQ, and others. Automated ChlF image processing consisted of image segmentation by mask application, background subtraction and feature extraction. The signals from all pixels of each segment were averaged at each given time point. MF refers to the measuring flash, and yellow arrows indicate the saturation pulses that transiently saturated the electron transport chain. Lss1, Lss2, and Lss3 represent actinic photon irradiance measurements taken at 170, 620, and 1070 μmol photons m-2 s-1, respectively. [file Image_1.JPEG]

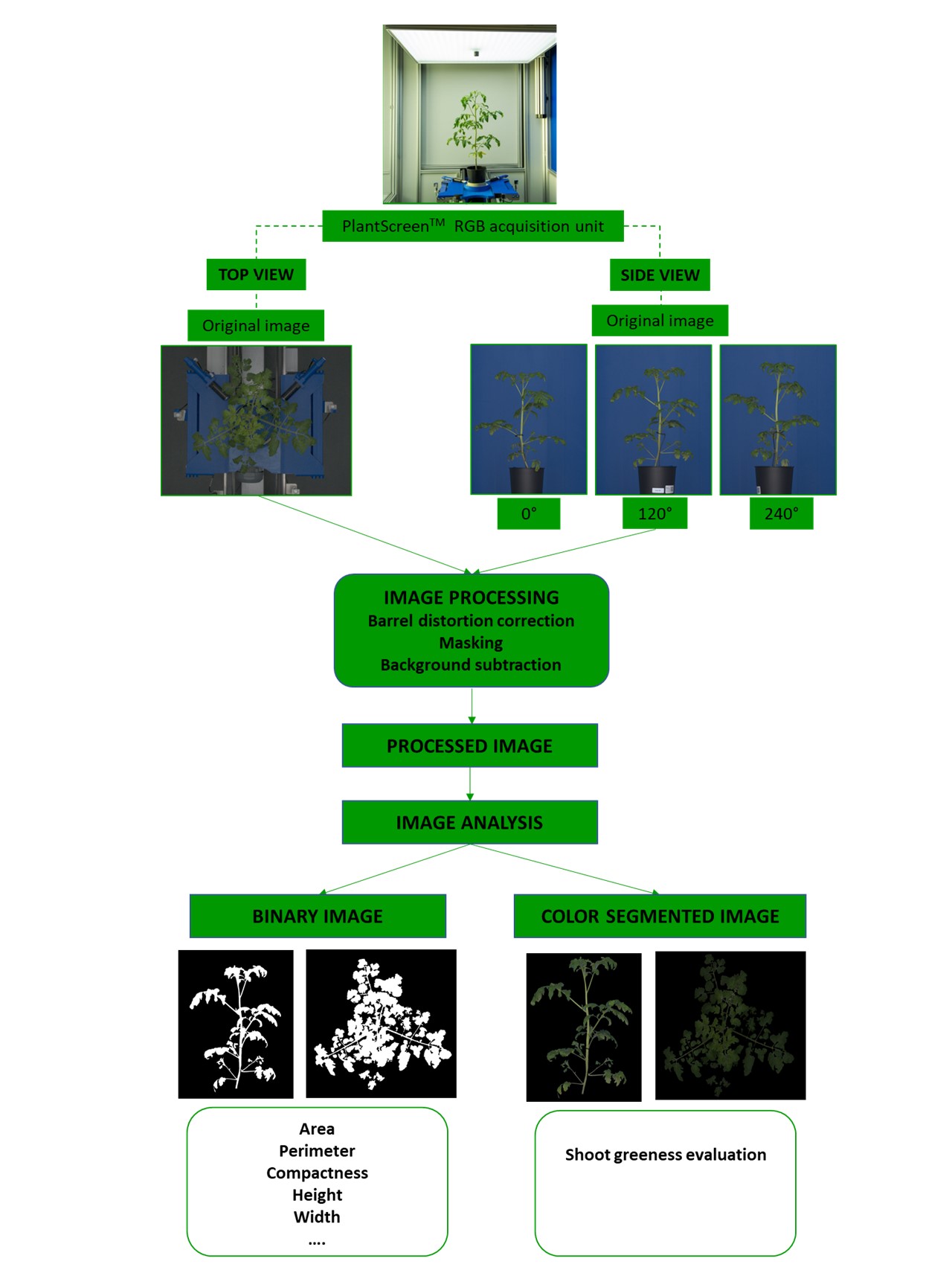

Supplement: FIGURE S2 — Schematic of top and side view RGB image processing. Original RGB images were automatically processed using the PlantScreenTM Analyzer software to correct for barrel distortion caused by the fisheye lens, subtract the background and crop to isolate the plants within the imaged area, producing a binary (black and white) image. The binary images represent the plant surface (white) and background (black). Non-plant pixels, such as pots, were automatically removed to extract only plant pixels. Morphological analysis was conducted after separating the background from the plant shoot tissue. To evaluate color of plant shoot, RGB images were color-segmented to extract the green hues. [file Image_2.JPEG]

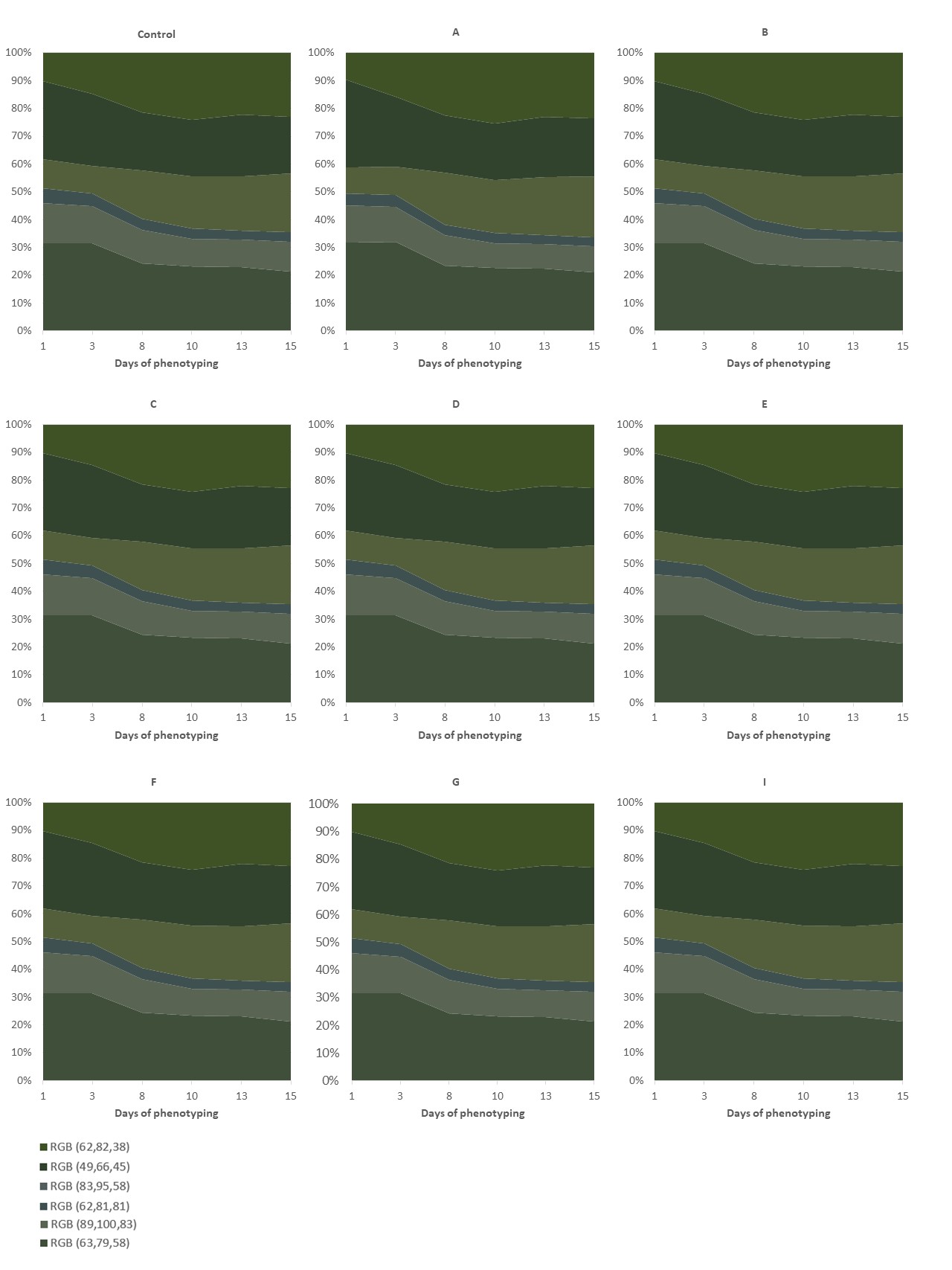

Supplement: FIGURE S3 — Variation in shoot colors of tomato plants. Dynamic relative changes in greenness hue abundance over the phenotyping period in control tomato plants and plants treated with protein hydrolysates (A–G, I). The six most representative color hues are shown in RGB color scale as percentage of the shoot area (pixel counts) of six biological replicates per treatment. [file Image_3.JPEG]
