# Supplementary material for: Understanding the Biostimulant Action of Vegetal-Derived Protein Hydrolysates by High-Throughput Plant Phenotyping and Metabolomics: A Case Study on Tomato
Source: Front Plant Sci. 2019 Feb 8;10:47. doi: 10.3389/fpls.2019.00047 (PMC6376207; doi:10.3389/fpls.2019.00047)
Supplement: TABLE S1 — Projected shoot area (PSA) of the tomato plants extracted from top view RGB images starting 3 days after the first PH application (day after transplanting, DAT = 8). Values are expressed as number of green pixels and represent the average of six biological replicates per treatment ± standard deviation. Within the same row and for the specified day different letters indicate significant difference according to one-way ANOVA post hoc Tukey’s test (p < 0.05). [file Table_1.DOCX]

**Suppl. Table 1** - Projected shoot area (PSA) of the tomato plants extracted from top view RGB images starting 3 days after the first PH application (day after transplanting, DAT = 8). Values are expressed as number of green pixels and represent the average of six biological replicates per treatment ± standard deviation. Within the same row and for the specified day different letters indicate significant difference according to one-way ANOVA post-hoc Tukey’s test (p<0.05).

| Treatment | DAT 8 | | DAT 10 | | DAT 13 | | DAT 15 | |
| --- | --- | --- | --- | --- | --- | --- | --- | --- |
| Control | 185466 ± 35826 | b | 258193 ± 63543 | b | 284354 ± 64892 | c | 372389 ± 65568 | b |
| A | 308192 ± 66640 | a | 401201 ± 86993 | ab | 591422 ± 80799 | a | 623942 ± 103262 | a |
| B | 279199 ± 22870 | ab | 352267 ± 36509 | ab | 522508 ± 45840 | ab | 596301 ± 85018 | ab |
| C | 212243 ± 30323 | ab | 329613 ± 42744 | ab | 402183 ± 52599 | bc | 493099 ± 52191 | ab |
| D | 296033 ± 55898 | ab | 386651 ± 75258 | ab | 531811 ± 101546 | ab | 614138 ± 108998 | a |
| E | 318470 ± 66810 | a | 427899 ± 96048 | a | 529265 ± 108641 | ab | 633845 ± 140093 | a |
| F | 277338 ± 84209 | ab | 349063 ± 87243 | ab | 451202 ± 96820 | abc | 558543 ± 131051 | ab |
| G | 248847 ± 63712 | ab | 350400 ± 91132 | ab | 501446 ± 140366 | ab | 595604 ± 165820 | ab |
| I | 235300 ± 34032 | ab | 298350 ± 27153 | ab | 471633 ± 27146 | abc | 511575 ± 76212 | ab |
