# Supplementary material for: Understanding the Biostimulant Action of Vegetal-Derived Protein Hydrolysates by High-Throughput Plant Phenotyping and Metabolomics: A Case Study on Tomato
Source: Front Plant Sci. 2019 Feb 8;10:47. doi: 10.3389/fpls.2019.00047 (PMC6376207; doi:10.3389/fpls.2019.00047)
Supplement: TABLE S2 — Projected shoot area (PSA) of the tomato plants extracted from multiple side view RGB images starting 3 days after the first PH application (day after transplanting, DAT = 8). Values are expressed as number of green pixels and represent the average of six biological replicates per treatment ± standard deviation. Within the same row and for the specified day different letters indicate significant difference according to one-way ANOVA post hoc Tukey’s test (p < 0.05). [file Table_2.DOCX]

**Suppl. Table 2 -** Projected shoot area (PSA) of the tomato plants extracted from multiple side view RGB images starting 3 days after the first PH application (day after transplanting, DAT = 8). Values are expressed as number of green pixels and represent the average of six biological replicates per treatment ± standard deviation. Within the same row and for the specified day different letters indicate significant difference according to one-way ANOVA post-hoc Tukey’s test (p<0.05).

| Treatment | DAT 8 | | DAT 10 | | DAT 13 | | DAT 15 | |
| --- | --- | --- | --- | --- | --- | --- | --- | --- |
| Control | 59923 ± 13370 | b | 92541 ± 17482 | b | 168051 ± 26977 | b | 198508 ± 38446 | b |
| A | 94001 ± 11175 | a | 143824 ± 13861 | a | 245531 ± 28656 | a | 308341 ± 31873 | a |
| B | 100127 ± 7961 | a | 149679 ± 9030 | a | 254850 ± 13077 | a | 290212 ± 29779 | ab |
| C | 76815 ± 15976 | ab | 110956 ± 14366 | ab | 186505 ± 30943 | ab | 227891 ± 22048 | ab |
| D | 87448 ± 12726 | ab | 126782 ± 15202 | ab | 213147 ± 28892 | ab | 268372 ± 27609 | ab |
| E | 88806 ± 16064 | ab | 136145 ± 20241 | ab | 238890 ± 34472 | ab | 289130 ± 49503 | ab |
| F | 86740 ± 19461 | ab | 129395 ± 30760 | ab | 227561 ± 54753 | ab | 272347 ± 65978 | ab |
| G | 83272 ± 25856 | ab | 122908 ± 33987 | ab | 205683 ± 54293 | ab | 254734 ± 65385 | ab |
| I | 73733 ± 15831 | ab | 114782 ± 14845 | ab | 201741 ± 25541 | ab | 262794 ± 35375 | ab |
