# Supplementary material for: Understanding the Biostimulant Action of Vegetal-Derived Protein Hydrolysates by High-Throughput Plant Phenotyping and Metabolomics: A Case Study on Tomato
Source: Front Plant Sci. 2019 Feb 8;10:47. doi: 10.3389/fpls.2019.00047 (PMC6376207; doi:10.3389/fpls.2019.00047)
Supplement: TABLE S3 — Width of the tomato plants extracted from multiple side view RGB images starting 3 days after the first PH application (day after transplanting, DAT = 8). Values are expressed as number of green pixels and represent the average of six biological replicates per treatment ± standard deviation. Within the same row and for the specified day different letters indicate significant difference according to one-way ANOVA post hoc Tukey’s test (p < 0.05). [file Table_3.DOCX]

**Suppl. Table 3** - Width of the tomato plants extracted from multiple side view RGB images starting 3 days after the first PH application (day after transplanting, DAT = 8). Values are expressed as number of green pixels and represent the average of six biological replicates per treatment ± standard deviation. Within the same row and for the specified day different letters indicate significant difference according to one-way ANOVA post-hoc Tukey’s test (p<0.05).

| Treatment | DAT 8 | | DAT 10 | | DAT 13 | | DAT 15 | |
| --- | --- | --- | --- | --- | --- | --- | --- | --- |
| Control | 625 ± 74 | c | 695 ± 77 | c | 835 ± 95 | c | 952 ± 110 | c |
| A | 758 ± 102 | a | 840 ± 82 | a | 1045 ± 79 | a | 1097 ± 75 | ab |
| B | 754 ± 81 | ab | 854 ± 97 | a | 1051 ± 99 | a | 1104 ± 53 | ab |
| C | 651 ± 88 | bc | 742 ± 82 | bc | 909 ± 83 | bc | 1002 ± 82 | bc |
| D | 742 ± 88 | ab | 827 ± 110 | a | 1022 ± 86 | a | 1076 ± 77 | ab |
| E | 739 ± 115 | ab | 858 ± 126 | a | 997 ± 91 | ab | 1133 ± 100 | a |
| F | 736 ± 116 | ab | 828 ± 157 | a | 970 ± 131 | ab | 1072 ± 153 | ab |
| G | 686 ± 142 | bc | 787 ± 124 | abc | 974 ± 155 | ab | 1056 ± 135 | abc |
| I | 663 ± 93 | bc | 782 ± 88 | abc | 996 ± 87 | ab | 1067 ± 120 | abc |
