# Supplementary material for: Understanding the Biostimulant Action of Vegetal-Derived Protein Hydrolysates by High-Throughput Plant Phenotyping and Metabolomics: A Case Study on Tomato
Source: Front Plant Sci. 2019 Feb 8;10:47. doi: 10.3389/fpls.2019.00047 (PMC6376207; doi:10.3389/fpls.2019.00047)
Supplement: TABLE S4 — Height of the tomato plants extracted from multiple side view RGB images starting 3 days after the first PH application (day after transplanting, DAT = 8). Values are expressed as number of green pixels and represent the average of six biological replicates per treatment ± standard deviation. Within the same row and for the specified day different letters indicate significant difference according to one-way ANOVA post hoc Tukey’s test (p < 0.05). [file Table_4.DOCX]

**Suppl. Table 4** - Height of the tomato plants extracted from multiple side view RGB images starting 3 days after the first PH application (day after transplanting, DAT = 8). Values are expressed as number of green pixels and represent the average of six biological replicates per treatment ± standard deviation. Within the same row and for the specified day different letters indicate significant difference according to one-way ANOVA post-hoc Tukey’s test (p<0.05).

| Treatment | DAT 8 | | DAT 10 | | DAT 13 | | DAT 15 | |
| --- | --- | --- | --- | --- | --- | --- | --- | --- |
| Control | 546 ± 67 | b | 734 ± 163 | bc | 930 ± 110 | d | 1174 ± 186 | c |
| A | 668 ± 62 | a | 824 ± 60 | ab | 1123 ± 82 | ab | 1337 ± 95 | ab |
| B | 687 ± 38 | a | 868 ± 61 | a | 1178 ± 74 | a | 1376 ± 94 | a |
| C | 671 ± 78 | a | 809 ± 44 | abc | 1048 ± 83 | bc | 1274 ± 57 | abc |
| D | 718 ± 77 | a | 899 ± 80 | a | 1203 ± 135 | a | 1387 ± 118 | a |
| E | 670 ± 51 | a | 812 ± 61 | ab | 1121 ± 72 | ab | 1335 ± 69 | ab |
| F | 694 ± 100 | a | 857 ± 139 | a | 1117 ± 151 | ab | 13222 ± 134 | ab |
| G | 692 ± 96 | a | 838 ± 118 | a | 1103 ± 139 | ab | 1333 ± 156 | ab |
| I | 566 ± 55 | b | 705 ± 88 | c | 978 ± 52 | cd | 1229 ± 98 | bc |
