# Supplementary material for: Understanding the Biostimulant Action of Vegetal-Derived Protein Hydrolysates by High-Throughput Plant Phenotyping and Metabolomics: A Case Study on Tomato
Source: Front Plant Sci. 2019 Feb 8;10:47. doi: 10.3389/fpls.2019.00047 (PMC6376207; doi:10.3389/fpls.2019.00047)
Supplement: TABLE S5 — Digital biomass of tomato plants treated with different protein hydrolysates starting 3 days after the first PH application (day after transplanting, DAT = 8). Values are expressed as number of green pixels and represent the average of six biological replicates per treatment ± standard deviation. Within the same row and for the specified day different letters indicate significant difference in digital biomass, according to one-way ANOVA post hoc Tukey’s test (p < 0.05). [file Table_5.DOCX]

**Suppl. Table 5 -** Digital biomass of tomato plants treated with different protein hydrolysates starting 3 days after the first PH application (day after transplanting, DAT = 8). Values are expressed as number of green pixels and represent the average of six biological replicates per treatment ± standard deviation. Within the same row and for the specified day different letters indicate significant difference in digital biomass, according to one-way ANOVA post-hoc Tukey’s test (p<0.05).

| Treatment | DAT 8 | | DAT 10 | | DAT 13 | | DAT 15 | |
| --- | --- | --- | --- | --- | --- | --- | --- | --- |
| Control | 26219341 ± 8887016 | b | 47651702 ± 15809090 | b | 90417958 ± 25638354 | b | 122435561 ± 35815347 | b |
| A | 52264360 ± 10850713 | a | 90992135 ± 15856605 | a | 189586844 ± 35368971 | a | 243509393 ± 38332729 | a |
| B | 47904900 ± 5653942 | ab | 84638470 ± 12135054 | a | 141658655 ± 8446546 | ab | 212774506 ± 38839091 | ab |
| C | 33574660 ± 10094184 | ab | 59562525 ± 9510096 | ab | 111651986 ± 21820459 | ab | 153092758 ± 22195505 | ab |
| D | 50343526 ± 11684467 | ab | 84031217 ± 17622181 | ab | 164085417 ± 36705056 | ab | 217636972 ± 42325628 | ab |
| E | 50269320 ± 13269985 | ab | 89461571 ± 23301168 | a | 174201900 ± 40624119 | ab | 232126717 ± 68680821 | a |
| F | 48826198 ± 16971615 | ab | 81055632 ± 27571693 | ab | 164014105 ± 53660635 | ab | 218211048 ± 75060581 | ab |
| G | 42691685 ± 18852229 | ab | 74504589 ± 30119149 | ab | 148228609 ± 56845130 | ab | 200866573 ± 80737148 | ab |
| I | 38361957 ± 7396873 | ab | 66422847 ± 10361545 | ab | 144633820 ± 16579042 | ab | 198209362 ± 33876201 | ab |

|  |
| --- |
