# Supplementary material for: Understanding the Biostimulant Action of Vegetal-Derived Protein Hydrolysates by High-Throughput Plant Phenotyping and Metabolomics: A Case Study on Tomato
Source: Front Plant Sci. 2019 Feb 8;10:47. doi: 10.3389/fpls.2019.00047 (PMC6376207; doi:10.3389/fpls.2019.00047)
Supplement: TABLE S6 — Variation in shoot colours of tomato plants treated with different protein hydrolysates at 15 days after transplanting. The values for 6 most representative colour hues are shown as percentage of the shoot area (pixel counts). Values represent the average of six biological replicates per treatment ± standard deviation. Within the same row and for the specified day different letters indicate significant difference according to one-way ANOVA post hoc Tukey’s test (p < 0.05). [file Table_6.DOCX]

**Suppl. Table 6 -** Variation in shoot colours of tomato plants treated with different protein hydrolysates at 15 days after transplanting. The values for 6 most representative colour hues are shown as percentage of the shoot area (pixel counts). Values represent the average of six biological replicates per treatment ± standard deviation. Within the same row and for the specified day different letters indicate significant difference according to one-way ANOVA post-hoc Tukey’s test (p<0.05).

| Treatment | RGB (63,79,58) | | RGB (89,100,83) | | RGB (83,95,58) | | RGB (49,66,45) | | RGB (62,81,81) | | RGB (62,82,38) | |
| --- | --- | --- | --- | --- | --- | --- | --- | --- | --- | --- | --- | --- |
| Control | 23 ± 3 | a | 11 ± 1 | a | 21 ± 3 | a | 19 ± 4 | a | 4 ± 0.3 | a | 23 ± 3 | a |
| A | 21 ± 2 | a | 9 ± 1 | a | 22 ± 1 | a | 21 ± 1 | a | 3 ± 0.3 | a | 24 ± 2 | a |
| B | 22 ± 1 | a | 10 ± 1 | a | 21 ± 2 | a | 21 ± 2 | a | 4 ± 0.1 | a | 23 ± 1 | a |
| C | 21 ± 2 | a | 12 ± 1 | a | 22 ± 4 | a | 19 ± 4 | a | 3 ± 0.4 | a | 24 ± 2 | a |
| D | 20 ± 2 | a | 11 ± 3 | a | 20 ± 2 | a | 21 ± 3 | a | 4 ± 0.4 | a | 24 ± 2 | a |
| E | 20 ± 1 | a | 11 ± 2 | a | 22 ± 2 | a | 20 ± 3 | a | 4 ± 0.2 | a | 23 ± 1 | a |
| F | 22 ± 3 | a | 11 ± 2 | a | 21 ± 1 | a | 22 ± 3 | a | 4 ± 0.3 | a | 21 ± 3 | a |
| G | 20 ± 2 | a | 12 ± 2 | a | 21 ± 1 | a | 20 ± 4 | a | 3 ± 0.2 | a | 23 ± 2 | a |
| I | 23 ± 3 | a | 9 ± 1 | a | 20 ± 2 | a | 22 ± 3 | a | 3 ± 0.1 | a | 22 ± 3 | a |
