# Supplementary material for: Understanding the Biostimulant Action of Vegetal-Derived Protein Hydrolysates by High-Throughput Plant Phenotyping and Metabolomics: A Case Study on Tomato
Source: Front Plant Sci. 2019 Feb 8;10:47. doi: 10.3389/fpls.2019.00047 (PMC6376207; doi:10.3389/fpls.2019.00047)
Supplement: TABLE S7 — Photosynthetic performance of tomato plants at 15 days after transplanting. Photosynthetic parameters deduced from kinetic chlorophyll fluorescence imaging on whole plant level in all protein hydrolysate treatments. Minimal fluorescence in dark-adapted state (Fo), maximum fluorescence in dark-adapted state (Fm), maximum quantum yield of PSII photochemistry for the dark-adapted (Fv/Fm), the photochemical quenching coefficient that estimates the fraction of open PSII reaction centers (qP), steady-state non-photochemical quenching (NPQ) and electron transport rate (ETR) were measured using the light curve protocol for tomato plants prior and upon PHs application. Values represent the average of six biological replicates per treatment ± standard deviation. Within the same row and for the specified day different letters indicate significant difference according to one-way ANOVA post hoc Tukey’s test (p < 0.05). Lss1, Lss2, and Lss3 represent actinic photon irradiance measurements taken at 170, 620, and 1070 μmol photons m-2s-1 PAR values, respectively. [file Table_7.DOCX]

**Suppl. Table 7 -** Photosynthetic performance of tomato plants at 15 days after transplanting. Photosynthetic parameters deduced from kinetic chlorophyll fluorescence imaging on whole plant level in all protein hydrolysate treatments. Minimal fluorescence in dark-adapted state (F_o_), maximum fluorescence in dark-adapted state (F_m_), maximum quantum yield of PSII photochemistry for the dark-adapted (F_v_/F_m_), the photochemical quenching coefficient that estimates the fraction of open PSII reaction centers (qP), steady-state non-photochemical quenching (NPQ) and electron transport rate (ETR) were measured using the light curve protocol for tomato plants prior and upon PHs application. Values represent the average of six biological replicates per treatment ± standard deviation. Within the same row and for the specified day different letters indicate significant difference according to one-way ANOVA post-hoc Tukey’s test (p<0.05). Lss1, Lss2 and Lss3 represent actinic photon irradiance measurements taken at 170, 620 and 1070 µmol photons m^-2^s^-1^ PAR values respectively.

| Lss 1 | | | | | | | | | | | | |
| --- | --- | --- | --- | --- | --- | --- | --- | --- | --- | --- | --- | --- |
| Treatment | **F_0_** | | **F_m_** | | **F_v_/F_m_** | | **qP** | | **NPQ** | | **ETR** | |
| Control | 67 ± 9 | a | 291 ± 27 | a | 0.77 ± 0.01 | a | 0.54 ± 0.16 | a | 0.60 ± 0.06 | a | 34 ± 9 | a |
| A | 73 ± 9 | a | 298 ± 30 | a | 0.76 ± 0.01 | a | 0.57 ± 0.14 | a | 0.71 ± 0.04 | a | 36 ± 8 | a |
| B | 67 ± 2 | a | 277 ± 7 | a | 0.76 ± 0.01 | a | 0.44 ± 0.19 | a | 0.72 ± 0.04 | a | 28 ± 12 | a |
| C | 68 ± 2 | a | 288 ± 18 | a | 0.76 ± 0.01 | a | 0.46 ± 0.18 | a | 0.66 ± 0.11 | a | 29 ± 12 | a |
| D | 68 ± 2 | a | 283 ± 5 | a | 0.76 ± 0.01 | a | 0.42 ± 0.17 | a | 0.71 ± 0.07 | a | 27 ± 11 | a |
| E | 68 ± 2 | a | 282 ± 13 | a | 0.76 ± 0.01 | a | 0.43 ± 0.17 | a | 0.70 ± 0.04 | a | 27 ± 11 | a |
| F | 66 ± 2 | a | 279 ± 9 | a | 0.76 ± 0.01 | a | 0.41 ± 0.05 | a | 0.67 ± 0.10 | a | 27 ± 11 | a |
| G | 69 ± 2 | a | 285 ± 8 | a | 0.76 ± 0.01 | a | 0.43 ± 0.18 | a | 0.70 ± 0.07 | a | 27 ± 11 | a |
| I | 76 ± 13 | a | 314 ± 35 | a | 0.76 ± 0.02 | a | 0.53 ± 0.31 | a | 0.60 ± 0.03 | a | 34 ± 10 | a |

| Lss 2 | | | | | | | | | | | | |
| --- | --- | --- | --- | --- | --- | --- | --- | --- | --- | --- | --- | --- |
| Treatment | **F_0_** | | **F_m_** | | **F_v_/F_m_** | | **qP** | | **NPQ** | | **ETR** | |
| Control | 61 ± 7 | a | 206 ± 10 | a | 0.70 ± 0.02 | a | 0.30 ± 0.16 | a | 1.10 ± 0.25 | ab | 55 ± 27 | a |
| A | 67 ± 7 | a | 214 ± 15 | a | 0.69 ± 0.02 | a | 0.33 ± 0.15 | a | 1.19 ± 0.30 | ab | 60 ± 25 | a |
| B | 62 ± 2 | a | 200 ± 3 | a | 0.69 ± 0.01 | a | 0.24 ± 0.09 | a | 1.42 ± 0.04 | a | 44 ± 16 | a |
| C | 63 ± 2 | a | 213 ± 12 | a | 0.70 ± 0.02 | a | 0.24 ± 0.09 | a | 1.25 ± 0.17 | ab | 45 ± 16 | a |
| D | 62 ± 2 | a | 205 ± 9 | a | 0.69 ± 0.01 | a | 0.23 ± 0.07 | a | 1.37 ± 0.08 | a | 42 ± 14 | a |
| E | 62 ± 2 | a | 207 ± 10 | a | 0.70 ± 0.01 | a | 0.22 ± 0.07 | a | 1.31 ± 0.08 | ab | 41 ± 13 | a |
| F | 61 ± 2 | a | 204 ± 14 | a | 0.70 ± 0.01 | a | 0.20 ± 0.07 | a | 1.29 ± 0.12 | ab | 40 ± 14 | a |
| G | 63 ± 2 | a | 206 ± 13 | a | 0.69 ± 0.01 | a | 0.23 ± 0.08 | a | 1.36 ± 0.09 | a | 42 ± 14 | a |
| I | 69 ± 11 | a | 221 ± 18 | a | 0.69 ± 0.03 | a | 0.37 ± 0.25 | a | 0.89 ± 0.35 | b | 66 ± 43 | a |

| Lss 3 | | | | | | | | | | | | |
| --- | --- | --- | --- | --- | --- | --- | --- | --- | --- | --- | --- | --- |
| Treatment | **F_0_** | | **F_m_** | | **F_v_/F_m_** | | **qP** | | **NPQ** | | **ETR** | |
| Control | 57 ± 6 | a | 168 ± 4 | a | 0.66 ± 0.03 | a | 0.23 ± 0.11 | a | 1.55 ± 0.23 | ab | 66 ± 27 | a |
| A | 63 ± 5 | a | 179 ±9 | a | 0.65 ± 0.02 | a | 0.25 ± 0.11 | a | 1.61 ± 0.29 | ab | 73 ± 26 | a |
| B | 58 ± 2 | a | 170 ± 1 | a | 0.65 ± 0.01 | a | 0.20 ± 0.06 | a | 1.87 ± 0.10 | a | 57 ± 16 | a |
| C | 60 ± 2 | a | 180 ± 12 | a | 0.67 ± 0.01 | a | 0.20 ± 0.05 | a | 1.66 ± 0.17 | ab | 57 ± 15 | a |
| D | 59 ± 2 | a | 172 ± 5 | a | 0.66 ± 0.01 | a | 0.19 ± 0.05 | a | 1.80 ± 0.06 | a | 55 ± 14 | a |
| E | 59 ± 2 | a | 175 ± 6 | a | 0.66 ± 0.01 | a | 0.18 ± 0.04 | a | 1.73 ± 0.07 | ab | 55 ± 12 | a |
| F | 57 ± 2 | a | 172 ± 9 | a | 0.66 ± 0.01 | a | 0.17 ± 0,05 | a | 1.71 ± 0.10 | ab | 52 ± 13 | a |
| G | 60 ± 1 | a | 174 ± 8 | a | 0.66 ± 0.01 | a | 0.19 ± 0.04 | a | 1.79 ± 0.05 | ab | 54 ± 13 | a |
| I | 63 ± 9 | a | 177 ± 11 | a | 0.64 ± 0.04 | a | 0.28 ± 0.17 | a | 1.35 ± 0.37 | b | 79 ± 44 | a |
